# Paul Glaucoma Implant following Congenital Cataract Surgery in a Pediatric Cohort

**DOI:** 10.3390/jcm13102914

**Published:** 2024-05-15

**Authors:** Angi Lizbeth Mendoza-Moreira, Anna Maria Voigt, Julia V. Stingl, Jasmin Rezapour, Felix Mathias Wagner, Alexander K. Schuster, Esther M. Hoffmann

**Affiliations:** Department of Ophthalmology, University Medical Center of the Johannes Gutenberg University Mainz, 55131 Mainz, Germany; amendoza@uni-mainz.de (A.L.M.-M.); annamaria.voigt@unimedizin-mainz.de (A.M.V.); julia.stingl@unimedizin-mainz.de (J.V.S.); jasmin.rezapour@unimedizin-mainz.de (J.R.); felix.wagner@unimedizin-mainz.de (F.M.W.); alexander.schuster@uni-mainz.de (A.K.S.)

**Keywords:** aphakia, congenital cataract surgery, Paul glaucoma implant, pediatric glaucoma, pediatric cohort

## Abstract

**Background**: The aim of this study was to evaluate the short-term efficacy and safety of the Paul Glaucoma Implant (PGI) in pediatric eyes diagnosed with glaucoma following congenital cataract surgery (GFCS). **Methods**: A retrospective, single-center, descriptive study was conducted on consecutive children diagnosed with GFCS who underwent PGI implantation between July 2022 and November 2023 at the University Medical Center Mainz. The primary outcome measure was the reduction in IOP at the last follow-up visit. **Results**: Ten eyes of nine children were included in the study. The mean follow-up time was 7.70 ± 4.22 months (4.68–10.72 months). At the end of the study follow-up, the mean (95% CI) reduction in IOP was −14.8 ± 8.73 mmHg (−8.56 to −21.04 mmHg, *p* < 0.001). At the last follow-up, 30.0% (3/10) of patients achieved an IOP (intraocular pressure) of ≥6 and ≤21 mmHg with a reduction in IOP of ≥25% without treatment, while 90.0% (9/10) achieved this target IOP regardless of glaucoma medication treatment. The mean number of antiglaucoma medications was significantly reduced from 3.50 (IQR = 1) to 2.0 (IQR = 2, *p* = 0.01), and the visual acuity logMAR improved from 1.26 ± 0.62 to 1.03 ± 0.48 (*p* = 0.04). Only one eye experienced numerical hypotony (4 mmHg) without choroidal detachment or anterior chamber shallowing within the first 24 h. No other adverse events were observed during the follow-up period. **Conclusions**: PGI implantation significantly lowered IOP and the number of antiglaucoma eye drops with a favorable safety profile in children diagnosed with GFCS, thereby achieving a high rate of qualified surgical success in the short term.

## 1. Introduction

Secondary glaucoma has been reported in 6% to 75.9% of children following congenital cataract surgery [1,2,3,4,5,6,7]. The incidence varies according to the criteria used in the studies [8]. Surgery can be necessary in 27–83% of glaucoma cases following congenital cataract surgery (GFCS) [9]. Spiess reported that up to 15% of children underwent surgery as the initial form of therapy, and 59% of patients who started on drops required subsequent surgical intervention in the following 3 months [8]. More than half of the eyes in published studies required two or more sequential surgical interventions [8]. Trabeculectomy, angle surgery, cyclodestructive laser treatment, and drainage implants are among the surgical treatments employed in these cases [3,4,7,8,9]. However, the insertion of a glaucoma drainage device (GDD) has shown better long-term control of IOP and tends to be the preferred technique over trabeculectomy [4,8,9,10,11,12]. The Ahmed Glaucoma Valve is the most common GDD used in pediatric GFCS [8,11,13]. The most common complication is hypotony, and it is often associated with athalamia/hypothalamia and/or choroidal detachment [8,12,13]. Tube exposure, tube-cornea touching, suprachoroidal hemorrhage, retinal detachment, and sterile infiltrates around the tube are also complications described within the Ahmed Glaucoma Valve for children with GFCS [9,13]. Especially in eyes with GFCS, there is a higher risk of hypotony or retinal detachment after surgery compared to primary congenital glaucoma, and the final visual acuity tends to be worse in the former group [10]. The Paul Glaucoma Implant (PGI; Advanced Ophthalmic Innovations PTE. LTD. Singapore) received the Conformité Européenne (CE) mark in Europe in 2018 [14]. The tube has an inner diameter of 0.127 mm, and it is supposed to produce less hypotony, tube exposure, and endothelial cell loss than other tube implants [14]. The intraluminal 6-0 Prolene suture prevents hypotony in the early postoperative period and allows for subsequent intraocular pressure (IOP) control through extraction during postoperative weeks 8–12 [15,16]. Few studies have evaluated the efficacy of PGI in the pediatric population [17,18]. Vallabh et al. reported the efficacy of PGI in four children with GFCS as part of a retrospective study involving 25 children aged 8 months to 16 years [18]. However, a subgroup analysis regarding the type of glaucoma was not conducted [18]. The present study aimed to report the short-term safety and effectiveness of PGI for glaucoma following congenital cataract surgery in a pediatric cohort, as this has not been previously reported.

## 2. Materials and Methods

We present a retrospective, single-center study of consecutive children with the diagnosis of glaucoma following congenital cataract surgery. 

The inclusion criteria comprised children under 18 years old who had undergone cataract surgery due to an isolated congenital cataract, without any other malformations or specified syndromes, and who underwent PGI (Advanced Ophthalmic Innovations PTE. LTD. Singapore) implantation between July 2022 and November 2023 at the University Medical Center Mainz. As our center serves as a reference center for childhood glaucoma in the country, we received children from various locations. It is important to note that we did not perform any cataract surgery on the subjects of our study. All the eyes were aphakic, and we did not perform any vitrectomy at the time of PGI implantation because we did not observe any clinical evidence of vitreous in the anterior chamber. All cataract surgeries were performed with vitrectomy and the removal of the posterior vitreous border membrane. Therefore, we do not routinely encounter vitreous in the anterior chamber. They were referred to us with a diagnosis of glaucoma, and many had undergone previous glaucoma surgeries at other hospitals. None of the previous implants were removed.

Pre- and postoperative data that were collected included intraocular pressure (IOP), a change in the number of antiglaucoma medications, a change in visual acuity, and the complete and qualified success with the incidence of adverse events. Intraocular pressure was measured using Applanation Tonometry during all office visits. 

### 2.1. Surgical Techniques

The same surgeon (EMH) performed all surgeries.

#### Paul-Glaucoma Implant Implantation

The disinfection of the eye to be operated on with povidone-iodine was performed, looping the cornea with 7.0 silk. The creation of a limbal-based conjunctival flap involved making an incision in the conjunctiva approximately 10 mm posteriorly from its insertion at the limbus. The quadrant for the incision was selected based on the available space and the quality of the conjunctiva and sclera observed intraoperatively by the surgeon. Blunt dissection forward and backward with scissors and further to the limbus with a crescent knife was performed. Cauterization Placement of two Merocel sponges soaked with 0.02% Mitomycin C (MMC). Duration: 3 min. Irrigation was performed with 30 mL of NaCl. The insertion of an intraluminal 6.0. Prolene suture into the tube was carried out. Paracentesis and the insertion of cohesive viscoelastic were performed until the intraocular pressure, assessed by palpation, reached approximately 20–25 mmHg. The insertion of the Paul implant was then conducted with the looping of the adjacent rectus muscles. The fixation of the Paul implant was performed with 8.0 Prolene anteriorly. Subsequent trimming of the tube and punctured with a 26-G needle parallel to the sclera to prepare a tunnel were carried out. The trimmed tube was inserted into the anterior chamber through the created tunnel. The anterior chamber form and pressure were adjusted with a balanced salt solution before the closure of the paracenteses. A 3 × 2 mm mesh material (Tutopatch^®^ or Tutoplast^®^) was placed on the tube before fixation with 10.0 nylon. The separate closure of Tenon’s capsule and conjunctiva (continuous) with 8/0 Vicryl was conducted, verifying the eye pressure by palpating the cornea with a cannula. The traction suture was then removed. Some viscoelastic was left in the anterior chamber. Finally, the subconjunctival injection of 4 mg of Dexamethasone, the application of ofloxacin ointment, and a bandage were conducted.

### 2.2. Outcomes

The primary outcome of this study was the intraocular pressure (IOP) at the end of the follow-up compared to the pre-operative baseline. Secondary outcome measures included the number of antiglaucoma eye drops (NOAM), visual acuity logMAR, complete and qualified success, and the incidence of adverse events. We evaluated three IOP targets according to the European Glaucoma Society guidelines as follows: IOP1, defined as ≤21 mmHg with a percentage reduction of ≥25% from the baseline; IOP2, defined as ≤18 mmHg with a percentage reduction of ≥30% from the baseline; and IOP3, defined as ≤15 mmHg with a percentage reduction of ≥40% from the baseline [19]. Complete success was achieved for each IOP target if the final IOP was attained without treatment and qualified success was achieved with treatment. Failure was considered if the IOP was greater than 21 mmHg or less than 6 mmHg or in cases of severe vision loss or the need for further surgical intervention to control the IOP. Intraluminal suture extraction was not classified as a “failure” event. Adverse events reported included numerical hypotony (IOP <6 mmHg with no associated complications), clinical hypotony (IOP <6 mmHg associated with choroidal detachment and/or anterior chamber depth reduction), choroidal detachment, severe anterior chamber shallowing (with iris-corneal touch), hyphema, endophthalmitis, IOP spike (elevation of 10 mmHg from preoperative IOP), corneal decompensation (corneal edema persisting for over 4 weeks), persistent uveitis (SUN grade >1 + cell in the anterior chamber persisting for 6 weeks), no light perception, leak, ptosis, diplopia, corneal Dellen, dysesthesia, iridodialysis, iris atrophy, device obstruction, device malposition, device migration, and device exposure [19]. 

### 2.3. Statistical Analysis

The statistical analysis was performed using IBM SPSS Statistics version 28 (IBM Corp, New York, NY, USA). Statistical significance was defined by a *p*-value less than 0.05. Data were tested for normal distribution using the Kolmogorov–Smirnov and Shapiro–Wilk test and were expressed as the number (percentage %), mean (standard deviation or 95% confidence interval), or median (interquartile range). Student’s paired *t*-test was employed to evaluate the change in IOP compared to the preoperative values, while the Wilcoxon signed-rank test was utilized to assess the number of glaucoma medications from the preoperative baseline to the last follow-up visit.

## 3. Results

Ten eyes diagnosed with glaucoma following congenital cataract surgery underwent Paul Glaucoma Implantation at our department from July 2022 to November 2023. The main baseline demographic and clinical characteristics of the whole cohort are detailed in Table 1 and summarized in Table 2. The mean age was 13.10 ± 3.64, years and the majority of the population was male (70.0%). All patients were aphakic. MMC was used in 8/10 eyes. The mean follow-up time was 7.70 ± 4.22 months (range, 4.68–10.72 months). Only two eyes had 12 months of follow-up. 

Case 2 and Case 10 underwent PGI surgery as their initial procedure due to failed angle surgery on the contralateral eye. Case 6 and Case 7 represented the same patient. The patient was satisfied with the outcomes of the initial PGI surgery on one eye and opted for the same procedure on the other eye due to its history of requiring two cyclocryocoagulations to control intraocular pressure (IOP). Case 8 had a history of failed trabeculotomy in the month prior to PGI implantation. The trabeculotomy had been performed due to glaucoma refractory to medication. Therefore, when the IOP was elevated, we chose PGI implantation.

**Figure 1:**
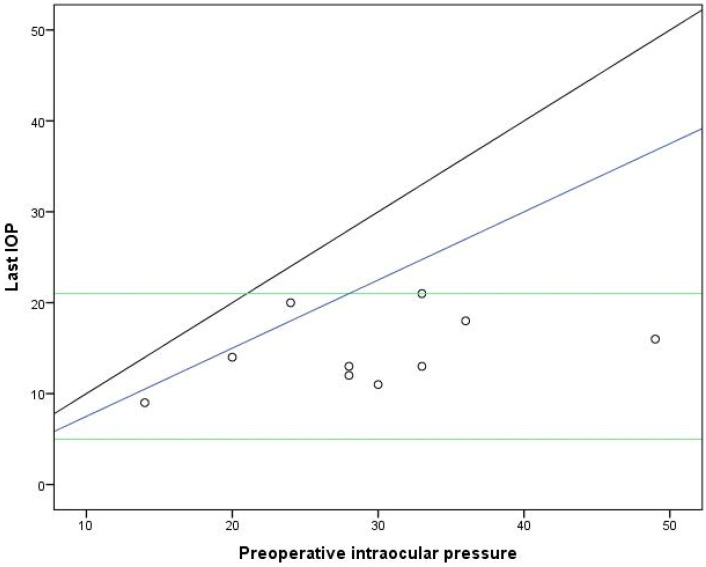
Intraocular pressure preoperative and at the last follow-up visit. Combined success criteria of ≤21 mmHg, ≥6 mmHg (horizontal lines), and ≥25% lowering of IOP (lower oblique line). The upper oblique line represents no change in IOP.

All eyes exhibited a reduction in IOP compared to the preoperative value (Figure 1). The baseline mean IOP was 29.50 ± 9.50 mmHg, which significantly reduced to 14.70 ± 3.95 mmHg at the last visit, with a mean change in IOP of 14.8 ± 8.73 mmHg (−8.56 to −21.04 mmHg) (*p* < 0.001, Student’s paired *t*-test), as shown in Table 3. 

**Figure 2:**
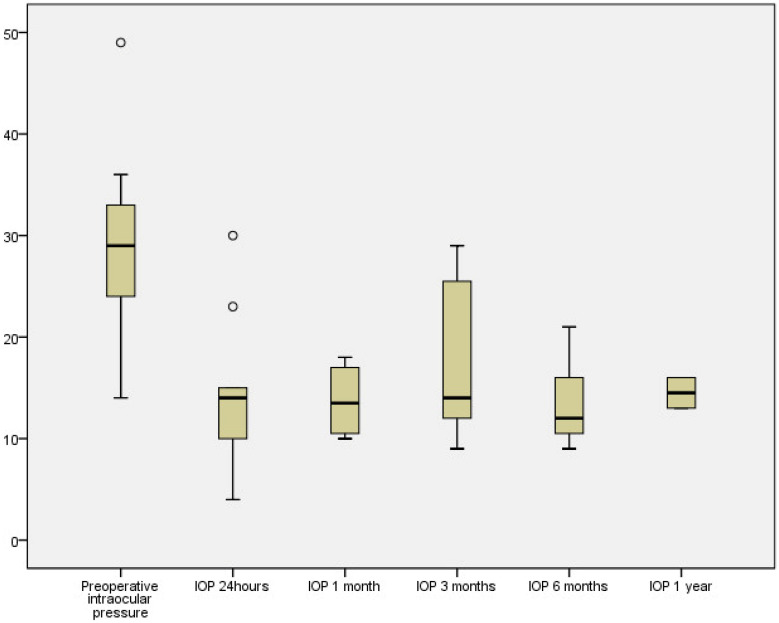
Intraocular pressure development. Number of eyes, 10, 9, 7, 7 and 2, preoperative, 24 h, and 1, 3, 6 and 12 months after, respectively.

Intraluminal Prolene was removed in only one patient at 140 days, with an IOP of 29 mmHg with two antiglaucoma medications. Figure 2 illustrates the IOP behavior at various time points. At the 24 h mark, two patients experienced ocular hypertension, likely due to the viscoelastic, which normalized within one day. There was a tendency for IOP to increase at 3 months when most patients began receiving antiglaucoma medication.

The median number of medications used preoperatively was 3.50 (IQR = 1), decreasing to 2.0 (IQR = 2) at the last follow-up visit. The mean change in the number of medications from the preoperative visit to the last visit showed a statistically significant reduction of −1.90 ± 1.45 (−0.86 to −2.94) (*p* = 0.01, Wilcoxon matched-pairs signed-rank test), as presented in Table 2. The mean preoperative visual acuity (VA) logMAR was 1.26 ± 0.62 and improved to 1.03 ± 0.48 at the last visit, with a mean change of 0.23 ± 0.31 (*p* = 0.04, Wilcoxon matched-pairs signed-rank test).

Table 4 illustrates the proportion of patients who attained complete and qualified success according to the three IOP targets outlined by the EGS.19. These criteria are notably stringent, especially in the context of secondary glaucoma following lens extraction. The proportion of complete success was low for each target IOP. Qualified success decreased as the target IOP decreased. In total, 80% of eyes achieved cumulative qualified success with a target of ≤18 mmHg and a reduction of ≥30%, while the proportion decreased to 40% with a target IOP ≤ 15 mmHg and a reduction of ≥40%. Failure was not reported. All patients achieved an IOP ≤21 mmHg at the last follow-up visit. No patient experienced severe vision loss or required further surgical intervention to control the IOP apart from the intraluminal suture extraction. 

Regarding the safety profile, only one eye experienced numerical hypotony (4 mmHg) without choroidal detachment or reduction in the anterior chamber depth within the first 24 h. This eye had a preoperative IOP of 30 mmHg while taking four antiglaucoma medications and a history of five cyclophotocoagulations. Its last recorded IOP at eight months was 11 mmHg without medication or intraluminal suture extraction. No other adverse events were observed during the follow-up period. 

## 4. Discussion

This study evaluated the initial outcomes of PGI in children with GFCS, focusing on intraocular pressure (IOP), the reduction in antiglaucoma medication usage, and its safety profile. We found a statistically significant reduction in postoperative IOP (−14.8 ± 8.73 mmHg) and the number of antiglaucoma medications (−1.90 ± 1.45), with 100% qualified surgical success at the last follow-up visit (mean follow-up time was 7.70 ± 4.22 months).

Our final mean IOP was higher than the mean IOP reported by Vallabh et al. at the end of their follow-up (13.2 ± 4.9 mmHg at 12 months, and for 24 months, the mean IOP was 11.8 ± 4.6 mmHg) [18]. One contributing factor to this difference is that Vallabh et al. removed the intraluminal suture in 9 out of 25 eyes, compared to only 1 out of 10 eyes in our study [18]. In pediatric cases, the removal of the intraluminal suture typically requires the use of general anesthesia. Our preference was to control IOP with antiglaucoma medication and only consider intraluminal suture removal when the IOP exceeded the personalized target despite maximal tolerable medication. Other factors contributing to the difference in IOP control included the tendency for eyes with GFCS to exhibit poorer IOP control and thicker corneas compared to those with PCG [10,20]. Thick corneas could result in false high IOP measures [20]. Therefore, IOP is generally accepted to be higher in children with GFCS [20]. In the study by Vallabh et al., only 4 out of 25 eyes had a diagnosis of GFCS [18]. The two most common diagnoses in their cohort were uveitic glaucoma and PCG [18]. This aligns with the findings of Elhusseiny et al., where one eye with GFCS reached an IOP of 18 mmHg, compared to the other two eyes with PCG, which had final IOPs of 10 and 11 mmHg, respectively [17].

The median number of medications at the last follow-up visit was higher than that reported by Vallabh et al. (1.0 ± 1.3 at 12 months and 1.3 ± 1.6 at 24 months) and Elhusseiny (2 out of 3 eyes without medication) [17,18]. This difference could also be related to intraluminal suture removal. We observed an improvement in VA, which was also noted by Vallabh [18]. However, this improvement was not statistically significant in the latter study [18]. No severe visual loss was reported in the children who received PGI [17,18]. 

Our results regarding qualified success (90%) were comparable to those of Vallabh et al., who reported an 84% success rate with a target IOP of <21 mmHg [18]. However, their definition of success did not include a percentage reduction in IOP from the baseline value, which is an additional strict criterion [18]. In the case series by Elhusseiny et al., the patient diagnosed with GFCS achieved an IOP of 18 mmHg with two medications at 6 months after intraluminal suture removal compared to a preoperative IOP of 24 mmHg, which met the criteria for qualified success [17]. We followed the criteria outlined by the EGS for publishing data from glaucoma trials [19]. However, it is important to note that these criteria are not specifically tailored for GFCS, which is a condition known for its challenging treatment and often poorer outcomes. Given the unique challenges faced by GFCS patients, we believe that strict adherence to surgical success criteria may not be appropriate for this patient population, and we opted for a broader definition of failure in our study to maintain comparability with other published studies. Thus, failure was defined as an IOP exceeding 21 mmHg or falling below 6 mmHg, or in cases of severe vision loss, the necessity for further surgical intervention to manage IOP. We encountered one patient who had an end-of-follow-up IOP of 20 mmHg, which represented only a 17% reduction from a preoperative IOP of 24 mmHg. Although this patient did not meet the EGS criteria for surgical success, the reduction in glaucoma medications from 4 to 0 suggests a favorable outcome. Therefore, we considered this patient neither a surgical success (according to EGS criteria) nor a failure.

No patient in our study or in Elhusseiny’s required further glaucoma surgery, in contrast to one patient in the study by Vallabh et al., who required trabeculotomy in both eyes [17,18]. Subsequent genetic testing revealed a heterozygous c.1109C > T p.(Pro370Leu) variant in the MYOC gene in this patient [18]. Additionally, two patients were deemed failures due to hypotony at 6 months after intraluminal suture removal [18]. We only had one case of numerical hypotony 24 h postoperatively and no other adverse events, in comparison to other studies of PGI in the pediatric population where, besides hypotony, a case of retinal detachment, vitreous entering the tube in an aphakic patient, scleral exposure, which subsequently scarred without further intervention, and keratoplasty graft failure were reported [17,18]. The follow-up period in Vallabh’s study is longer than ours and Elhusseiny’s [17,18]. They followed patients for 24 months, which allowed for the detection of more surgical failures and late adverse events [18].

Currently, only two published studies have evaluated the use of PGI in the pediatric population [17,18]. Our study represents the first to report the outcomes of PGI in children with GFCS, which is known to be very resistant to therapeutic approaches. GFCS is often challenging to manage and is generally associated with a poor prognosis in terms of IOP and VA [9,10]. These patients usually require more medication post-surgery for long-term control compared to PCG [10]. We did not routinely encounter vitreous in the anterior chamber because all cataract surgeries were performed with vitrectomy with the removal of a posterior vitreous border membrane. However, over time, the posterior vitreous may become loose and may incarcerate the tube in aphakic eyes where there was aphakic without vitrectomy at the time of the GDD implantation [21,22,23,24]. Vigilant observation for incarceration is required over the patient’s lifetime, along with appropriate treatment [21,22,23,24].

## 5. Conclusions

Our findings suggest that PGI is a viable and safe option for these patients. PGI significantly reduces both IOP and the need for antiglaucoma medications, leading to a high qualified success rate in children with GFCS and a favorable safety profile. While we acknowledge the limitations of our current study, including its small sample size, retrospective design, and short follow-up period, we anticipate further insights from the long-term follow-up of this cohort. Future research endeavors should focus on larger sample sizes, prospective methodologies, and extended follow-up periods to comprehensively evaluate the efficacy and safety of PGI in children with glaucoma following congenital cataract surgery. Additionally, comparative studies comparing PGI with other glaucoma drainage devices are warranted. Given the ethical challenges associated with conducting randomized controlled trials (RCTs) in children, high-quality retrospective studies can also provide valuable insights into the effectiveness and safety of new surgical techniques in this population. 

## Figures and Tables

**Table 1:** Baseline and postoperative clinical information of the whole cohort.

Eyes	Gender	Age at the Time of PGI Implantation	Status of the Lens	Previous Glaucoma Surgeries	Laterality	Place of the PGI Implantation	Preoperative Visual Acuity logMAR	Preoperative IOP	Preoperative Glaucoma Medications	IOP at the Final Follow-Up	Glaucoma Medication at the Final Follow-Up	Visual Acuity at the Final Follow-Up logMAR	Duration of the Follow-Up (Months)
1	Male	11	Aphakic	Trabeculectomy, Bleb Revision 2 times, XEN, Ahmed Valve, Cyclocryocoagulation	Right	Temporal inferior	1.3	28	3	12	2	1.3	4
2	Female	12	Aphakic	None	Left	Temporal superior	1.6	20	3	14	2	0.7	10
3	Female	12	Aphakic	Trabeculotomy, Ahmed Valve, Bleb-Revision, Cyclocryocoagulation	Right	Temporal inferior	2.28	33	4	21	2	1.7	7
4	Male	13	Aphakic	Cyclocryocoagulation	Left	Temporal superior	0.80	24	4	20	0	0.6	4
5	Male	14	Aphakic	Trabeculectomy, Ahmed Valve	Left	Nasal inferior	0.80	36	4	18	2	0.8	5
6	Male	17	Aphakic	Cyclophotocoagulation 5 times	Right	Nasal superior	0.7	30	4	11	0	0.7	8
7	Male	17	Aphakic	Cyclophotocoagulation 2 times	Left	Temporal superior	0.6	14	3	9	0	0.6	7
8	Male	16	Aphakic	Trabeculotomy	Left	Temporal superior	1.30	28	0	13	0	1.0	16
9	Female	6	Aphakic	Trabeculotomy	Left	Temporal superior	2.28	33	3	13	3	1.98	3
10	Male	11	Aphakic	None	Left	Temporal superior	0.9	49	4	16	2	0.9	13

**Table 2:** Summary of demographic and clinical characteristics of the 10 eyes in the study population.

Characteristics	Data
Age, years	
Mean (SD)	13.10 (3.64)
95% CI	10.50 to 15.70
Gender, n (%)	
Male	7 (70.0)
Female	3 (30.0)
Laterality, n (%)	
Right	3 (30.0)
Left	7 (70.0)
Ethnic group, n (%)	
North European	5 (50.0)
South European	3 (30.0)
South Asian	1 (10.0)
Middle East	1 (10.0)
Previous glaucoma surgery, n (%)	
Yes	8 (80.0)
No	2 (20.0)
Number of previous glaucoma surgeries	
Mean (SD)	2.40 (2.07)
95% CI	0.92 to 3.88
Previous glaucoma surgery, n (%)	
Trabeculectomy	2 (8.33)
Trabeculotomy	3 (12.5)
Glaucoma drainage device	3 (12.5)
XEN-Stent	1 (4.16)
Open Revision	3 (12.5)
Cyclocryocoagulation	4 (16.66)
Cyclophotocoagulation	8 (33.33)
Visual acuity, logMAR	
Mean (SD)	1.26 (0.62)
95% CI	0.81 to 1.70
IOP, mm Hg	
Mean (SD)	29.50 (9.50)
95% CI	22.70 to 36.30
Number of preoperative drops	
Median (IQR)	3.50 (1)
Oral carbonic anhydrase inhibitor, n (%)	
Yes	3 (30.0)
No	7 (70.0)

SD, standard deviation. IQR, interquartile range. IOP, intraocular pressure.

**Table 3:** Overview of the evolution of intraocular pressure (IOP) and number of antiglaucoma medications (NOAM) from the baseline for eyes.

IOP End follow-up	Mean ± SD (95% CI) absolute difference from baseline	*p* ^a^
	−14.80 ± 8.73 (−8.56 to −21.04)	<0.001
NOAM End follow-up	Mean ± SD (95% CI) absolute difference from baseline	*p* ^b^
	−1.90 ± 1.45 (−0.86 to −2.94)	0.01
Visual acuity (logMAR) End follow-up	Mean ± SD (95% CI) absolute difference from baseline	*p* ^a^
	−0.23 ± 0.31(−0.09 to −0.45)	0.04

^a^ Student’s paired *t*-test. ^b^ Wilcoxon signed rank test. SD, standard deviation. IOP, intraocular pressure.

**Table 4:** Complete and qualified. The data are expressed as the number of eyes (%).

	Complete Success	Qualified Success
IOP ≥6 mmHg ≤21 mmHg and a percentage reduction of ≥25%	3/10 (30.0)	9/10 (90.0)
IOP ≥6 mmHg ≤18 mmHg and a percentage reduction of ≥30%	3/10 (30.0)	8/10 (80.0)
IOP ≥6 mmHg ≤15 mmHg and a percentage reduction of ≥40%	2/10 (20.0)	4/10 (40.0)

## Data Availability

The data presented in this study are available on request from the corresponding author. The data are not publicly available due to them containing information that could compromise the privacy of research participants.
